# Endogenous Murine BST-2/Tetherin Is Not a Major Restriction Factor of Influenza A Virus Infection

**DOI:** 10.1371/journal.pone.0142925

**Published:** 2015-11-13

**Authors:** Sarah L. Londrigan, Michelle D. Tate, Emma R. Job, Jessica M. Moffat, Linda M. Wakim, Christopher A. Gonelli, Damien F. J. Purcell, Andrew G. Brooks, Jose A. Villadangos, Patrick C. Reading, Justine D. Mintern

**Affiliations:** 1 Department of Microbiology and Immunology, Peter Doherty Institute for Infection and Immunity, The University of Melbourne, Parkville, Victoria, 3010, Australia; 2 Centre for Innate Immunity and Infectious Diseases, MIMR-PHI Institute of Medical Research, Clayton, Victoria, 3168, Australia; 3 Monash University, Clayton, Victoria, 3168, Australia; 4 Department of Biochemistry and Molecular Biology, The University of Melbourne, Bio21 Molecular Science and Biotechnology Institute, 30 Flemington Rd, Parkville, Victoria, 3010, Australia; 5 WHO Collaborating Centre for Reference and Research on Influenza, Victorian Infectious Diseases Reference Laboratory, at the Peter Doherty Institute for Infection and Immunity, Melbourne, Victoria, 3000, Australia; Helmholtz Zentrum Muenchen—German Research Center for Environmental Health, GERMANY

## Abstract

BST-2 (tetherin, CD317, HM1.24) restricts virus growth by tethering enveloped viruses to the cell surface. The role of BST-2 during influenza A virus infection (IAV) is controversial. Here, we assessed the capacity of endogenous BST-2 to restrict IAV in primary murine cells. IAV infection increased BST-2 surface expression by primary macrophages, but not alveolar epithelial cells (AEC). BST-2-deficient AEC and macrophages displayed no difference in susceptibility to IAV infection relative to wild type cells. Furthermore, BST-2 played little role in infectious IAV release from either AEC or macrophages. To examine BST-2 during IAV infection *in vivo*, we infected BST-2-deficient mice. No difference in weight loss or in viral loads in the lungs and/or nasal tissues were detected between BST-2-deficient and wild type animals. This study rules out a major role for endogenous BST-2 in modulating IAV in the mouse model of infection.

## Introduction

BST-2 (tetherin; CD317, HM1.24) is a host cell protein of importance to viral immunity. BST-2 prevents newly generated viral particles from being released from the infected cell by forming a "tether" that retains virions at the cell surface. Viral tethering by BST-2 was first identified for human immunodeficiency virus [1, 2] with subsequent studies showing activity against many enveloped viruses [3–6]. In addition to viral tethering, BST-2 can exert other immunomodulatory functions during viral infection [7, 8]. Specifically, BST-2 has been shown to modulate type 1 interferon (IFN) production [9, 10] and trigger NFκB activation [11–13]. Consequently, BST-2 is a molecule of significant interest in host viral defense.

Influenza viruses belong to the *Orthomyxoviridae* family of enveloped viruses and are an important cause of respiratory disease worldwide. Type A influenza virus (IAV) is the major etiological agent capable of causing epidemics and pandemics in humans. Host anti-viral restriction factors have been described that can limit intracellular replication of IAV. Examples include interferon (IFN)-inducible transmembrane-3 [14, 15], viperin [16] and myxovirus resistance gene A [17, 18]. Currently, whether BST-2 acts to restrict the release of infectious IAV is controversial. Initial reports indicated that BST-2 limited release of IAV virus-like particles (VLP) [19, 20], as well as release of infectious IAV [21–23]. In contrast, several studies reported that BST-2 did not restrict IAV release from infected cells [19, 24, 25]. Overall, studies investigating the role of BST-2 during IAV infection have utilized cell lines engineered to overexpress or suppress BST-2. Differences in BST-2 expression levels, the contribution of transformed cell lines and/or expression of BST-2 in heterologous cell lines may be important factors contributing to the conflicting data published to date. Hence, further studies are required to clarify the impact of BST-2 during IAV infection, using primary cells susceptible to IAV infection where physiologically relevant levels of endogenous BST-2 are expressed. To date, one study has examined the role of BST-2 during influenza virus infection *in vivo*. Surprisingly, BST-2-deficient mice infected with influenza B virus (IBV) displayed a modest, but significant, reduction in lung viral titers at day 3 post-infection, although no significant differences were detected at day 6 [7]. This finding is paradoxical to the proposed role of BST-2 in limiting viral release, and so far remains largely unexplained.

In the airways, primary alveolar epithelial cells (AEC) and macrophages represent two of the major cell types susceptible to IAV infection [26]. Herein, we have compared AEC and macrophages isolated from wild-type and BST-2-deficient mice [7], for their susceptibility to IAV infection and their ability to support productive IAV replication *in vitro*. In addition, we have compared weight loss and viral replication following intranasal infection of wild type or BST-2-deficient mice with IAV. Together, our data indicate that endogenous BST-2 does not play a major role in host restriction of IAV in the mouse model of infection.

## Materials and Methods

### Mice

C57BL/6 and BST-2-deficient mice (kindly provided by M. Colonna, Washington University [7]) were bred and housed in specific pathogen-free conditions at the Bio21 Animal House Facility, The University of Melbourne. Mice (female and male) 6–10 weeks of age were used in experiments conducted in accordance with guidelines provided by National Health and Medical Research Council of Australia. Experimental procedures were approved by the Animal Ethics Committees at the University of Melbourne (Application 1112261). Studies comply with Animal Research: Reporting In Vivo Experiment guidelines (S1 Checklist).

### Cell lines, primary macrophages and primary alveolar epithelial cells

Cell lines used in this study included Madin-Darby canine kidney (MDCK) cells (American Type Culture Collection, ATCC), LA-4 mouse lung epithelial cells and the RAW264.7 macrophage cell line (ATCC). Resident peritoneal exudate macrophages were obtained from mice as previously described [27]. Macrophages were seeded into 8-well glass chamber-slides (Lab-Tek), incubated for 2 hours at 37°C and cell monolayers were washed to remove non-adherent cells. The next day, any remaining non-adherent cells were removed and the adherent macrophages used in virus infection assays. Mouse primary lung epithelial cells were prepared as previously described [28]. Briefly, lungs from mice were digested in 1.5 mg/ml Pronase (Roche, USA), 0.1 mg/ml DNase I (Sigma-Aldrich, USA) for 1 hour at 37°C in 5% CO_2_. Single cell suspensions were incubated with purified rat anti-mouse CD45 antibody (BD Biosciences, USA) and epithelial cells negatively enriched with BioMag goat anti-rat immunoglobulin-coupled magnetic beads (Qiagen, USA). Cells were cultured on collagen-coated (MP Biomedicals, USA) plates. To confirm alveolar epithelial cell purity, monolayers were detached with 3 mM EDTA and cells stained with mouse anti-EpCAM, anti-podoplanin (AEC type I) and anti-CD74 (AEC type II) antibodies and examined by flow cytometry.

### Viruses

The representative IAV laboratory strain used in this study was HKx31, a reassortant of A/PR8/34 (PR8, H1N1) with A/Aichi/2/68 (H3N2) bearing the H3N2 surface glycoproteins. In some experiments A/Brazil/11/78 (Brazil/78; H1N1) and A/Solomon Islands/3/2006 (Sol Is/06; H1N1) were used as representative seasonal strains. Sol Is/06 was obtained from the World Heath Organization Collaborating Centre for Reference and Research on Influenza, Melbourne, Australia. Virus was amplified in the allantoic cavity of 10-day-old embryonated hen’s eggs and titrated on MDCK cells by standard plaque assay [29].

### Virus infection of mice

Groups of 5 mice were lightly anaesthetized (methoxyflurane) and infected with either 10^2^ or 10^4^ PFU of HKx31 via the intranasal route. Mice were weighed daily and assessed for signs of clinical disease. Animals that had lost ≥ 20% of their original body weight were euthanized by CO_2_ inhalation followed by cervical dislocation. No adverse events occurred during these experiments. Lungs and nasal tissues were removed, homogenized and clarified by centrifugation. Titers of infectious virus in tissue homogenates were determined by standard plaque assays [29].

### Virus infection assays

Mouse peritoneal macrophages and primary lung epithelial cells were infected with IAV and the percentage of IAV-infected cells determined as described previously for epithelial cells [30] and macrophages [31]. Briefly, cells were incubated with IAV in serum-free media for 1 hour at 37°C. Virus inoculum was removed and cells incubated at 37°C in serum-free media. IAV-infected cells were fixed with 80% vol/vol acetone at the indicated time points post-infection and stained using monoclonal antibody (mAb) MP3.10g2.1C7 (WHO Collaborating Centre for Reference and Research on Influenza, Melbourne, Australia) specific for IAV nucleoprotein. Virus-infected cells were co-stained with 4',6-diamidino-2-phenylindole (DAPI) or propidium iodide (PI). A minimum of 200 cells were scored for each sample.

### Virus growth assays

Cells were infected with IAV as described above. As trypsin counteracts BST-2 anti-viral activity [32], this was omitted in the infection media, thereby limiting IAV replication to a single round. Cell supernatants were collected at the indicated time-points post-infection, and incubated with 4 μg/ml L-(tosylamido-2-phenyl) ethyl chloromethyl ketone (TPCK)-treated trypsin (Sigma Aldrich) for 30 minutes at 37°C to facilitate cleavage of the viral hemagglutinin [33] before the titers of infectious virus were determined by standard plaque assay on MDCK cells [29].

### BST-2 expression assays

BST-2 expression was determined by staining with anti-BST-2 (clone 120G8) mAb in conjunction with flow cytometric analysis. Briefly, cells were pre-incubated with 2.4G2 mAb to block non-specific staining via Fc receptors, then stained with rat anti-BST-2 mAb conjugated to FITC (generated in house) or rat IgG1k-FITC isotype control antibody (Biolegend). In some experiments, BST-2 expression was monitored post-infection with IAV (as described above) at the indicated virus doses and time-points. Cells were treated with 1000 international units (IU)/ml recombinant murine IFNα (R&D Systems) as a control to upregulate BST-2 surface expression.

## Results

### BST-2 expression is upregulated on murine macrophages but not alveolar epithelial cells in response to IAV infection

**Fig 1:**
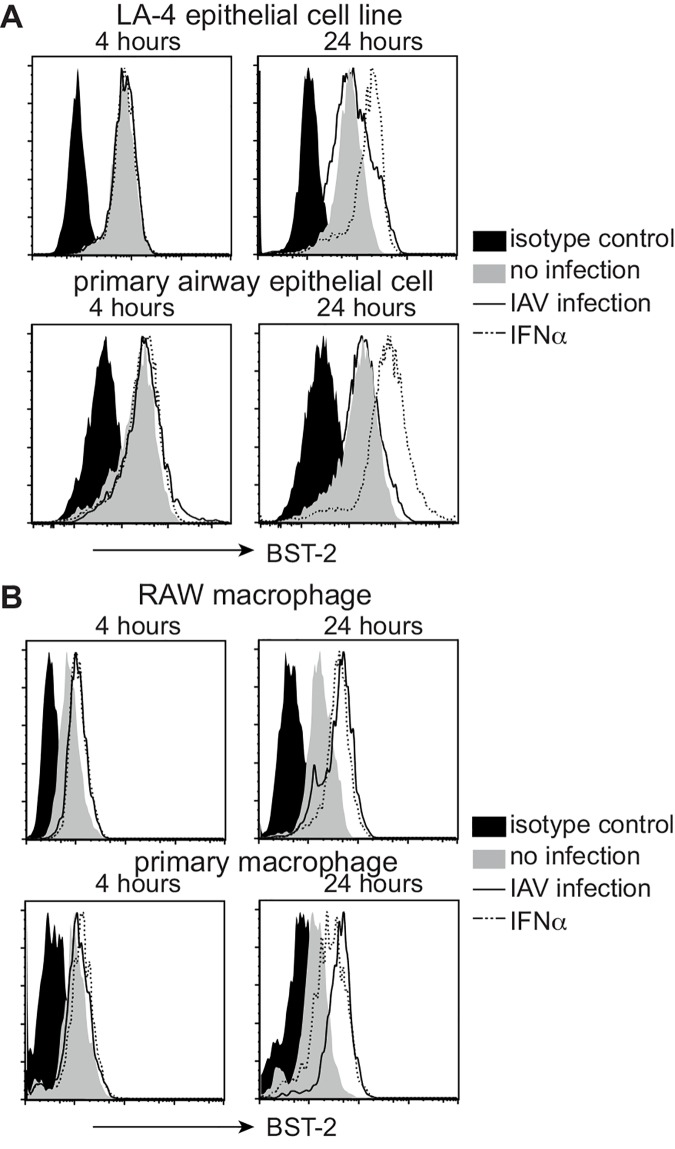
BST-2 expression is upregulated on murine macrophages but not alveolar epithelial cells in response to influenza A virus. Monolayers of (A) the LA-4 AEC line and primary AEC, or (B) RAW264.7 macrophages and primary macrophages were incubated (i) with a MOI of 5 (HKx31) for 1 hour at 37°C and washed to remove excess virus (IAV infection, solid black line), (ii) in 1000 IU/ml recombinant mouse IFNα (dashed line) or (iii) in media alone (no infection, grey histogram). Cells were then incubated at 37°C for a total of 4 or 24 hours and levels of cell-surface BST-2 determined by flow cytometry. For each cell type, the isotype control (solid black histograms) is shown for ‘no infection’ cells only but is representative of profiles obtained using IAV-infected and IFNα-treated cells. Data are representative of 3 independent experiments.

First, flow cytometry was used to examine BST-2 expression by murine AEC and macrophages that were uninfected, or had been infected 4 and 24 hours previously with IAV strain HKx31 (H3N2). BST-2 expression was analysed using the LA-4 AEC line and primary AEC, as well as using the RAW264.7 macrophage cell line and primary macrophages. Peritoneal exudate macrophages were used given the difficulty in obtaining sufficient numbers of alveolar macrophages via bronchoalveolar lavage (BAL). Note that peritoneal and alveolar macrophages exhibit similar susceptibility and ability to support IAV infection [34, 35]. For epithelial cells, uninfected LA-4 cells and primary AEC expressed cell-surface BST-2, however levels did not increase further following IAV infection. Of interest, culture of AEC in the presence of IFNα did result in upregulation of cell-surface BST-2 (Fig 1A). In contrast to epithelial cells, macrophages upregulated cell-surface BST-2 in response to IAV and levels were similar (RAW264.7), or higher (primary cells), than those elicited in response to IFNα (Fig 1B).

### BST-2 does not modulate susceptibility to IAV infection or release of newly synthesized virions from murine AEC and macrophages

**Fig 2:**
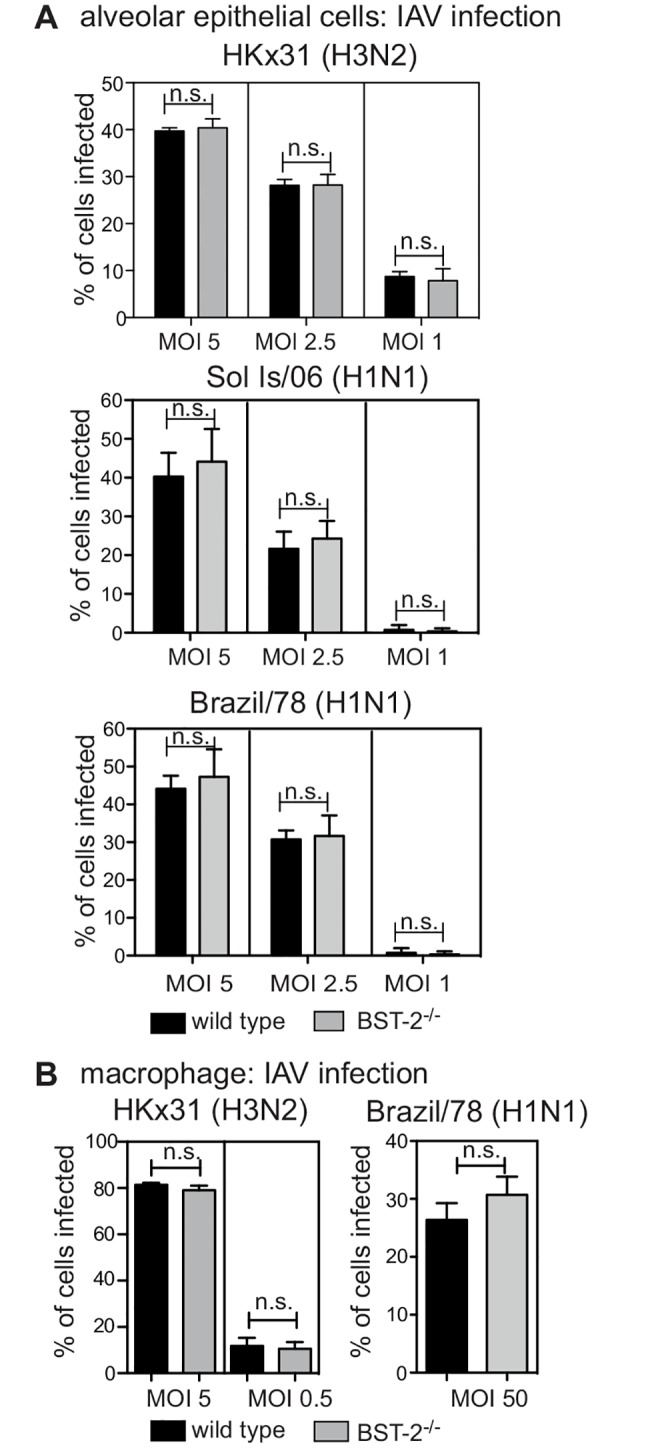
BST-2 expression does not modulate IAV susceptibility of murine epithelial cells and macrophages to IAV infection. (A) Primary AEC or (B) macrophages isolated from BST-2 wild type (WT) and BST-2-deficient (BST-2^-/-^) mice were incubated with the indicated MOI and strain of IAV for 1 hour at 37°C, washed to remove excess virus and cultured as indicated. Monolayers were fixed at 8 hours post-infection before staining by immunofluorescence to detect newly synthesized viral NP. Data show the mean (± 1 SD) pooled from 3 independent experiments. n.s. = no significant difference, *p* = > 0.05, two-way ANOVA followed by Bonferroni analysis.

Next, we examined the impact of BST-2 on the susceptibility of primary AEC and macrophages to IAV infection using cells from wild type (WT) and BST-2-deficient animals. We confirmed expression patterns of BST-2 by flow cytometry for primary macrophages isolated from BST-2^+/+^, BST-2^+/-^ and BST-2^-/-^ mice (S1 Fig). Next, cells isolated from WT or BST-2-deficient mice were inoculated with IAV at different multiplicities of infection (MOI) and the percentage of IAV-infected cells was determined 6–8 hours later by detection of newly synthesized viral nucleoprotein (NP) [35]. Viral NP was not detected at 2 hours post infection, indicating its presence at later time points was due to newly synthesized viral protein and not input virus (S2 Fig). Overall, the percentage of IAV-infected AEC did not differ in the presence or absence of BST-2 at any MOI tested for the IAV laboratory strain HKx31, or the representative seasonal strains Brazil/78 and Sol Is/06 (Fig 2A). Similar to AEC, macrophages exhibited equivalent susceptibility to IAV infection regardless of the presence or absence of BST-2 for both the HKx31 and Brazil/78 strains (Fig 2B). The Sol Is/06 IAV strain did not efficiently infect primary murine macrophages, and therefore was not assessed (data not shown).

**Fig 3:**
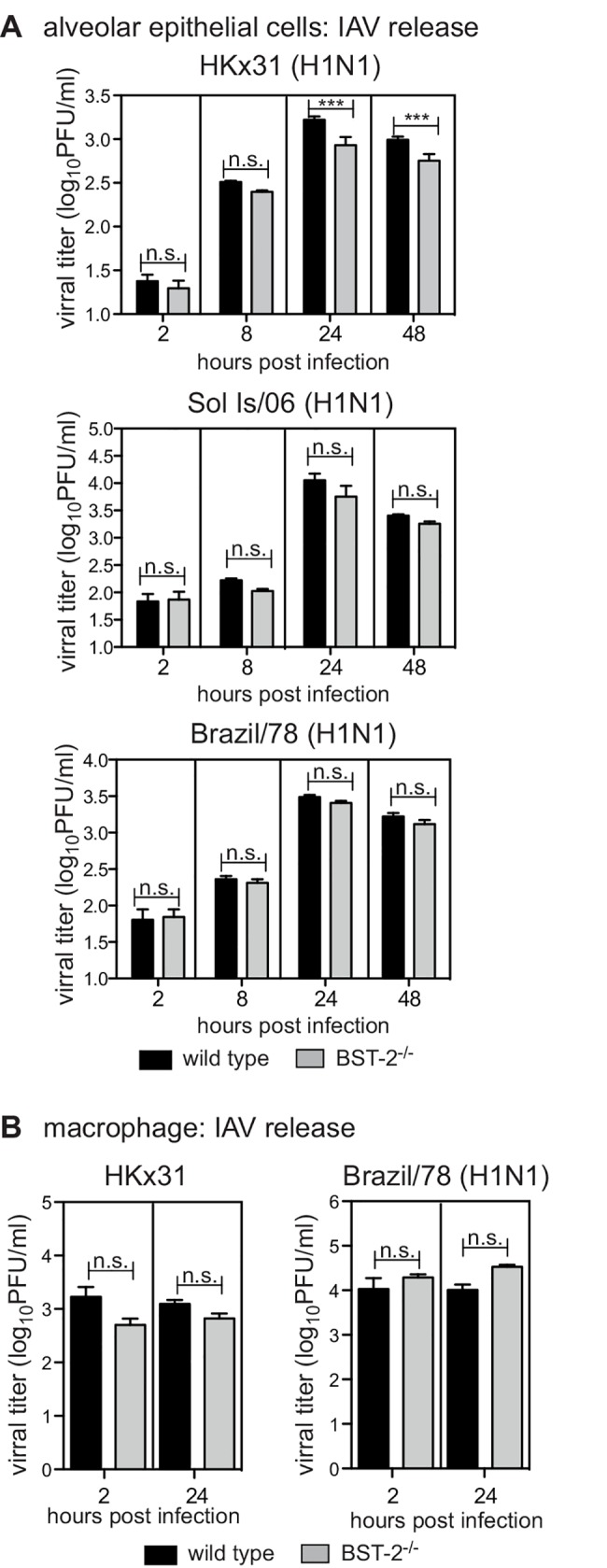
BST-2 expression does not modulate IAV release from murine epithelial cells and macrophages following IAV infection. (A) Primary AEC or (B) macrophages isolated from BST-2 wild type (WT) and BST-2-deficient (BST-2^-/-^) mice were incubated with the indicated strain of IAV for 1 hour at 37°C, washed to remove excess virus and cultured. AEC were infected at a MOI of 1 of HKx31, Brazil/78 and Sol Is/06 and macrophages infected at a MOI 5 for HKx31 and a MOI of 50 for Brazil/78. Culture supernatants were removed at 2 hours, or at 8, 24 or 48 hours as indicated, clarified by centrifugation and titres of infectious virus were determined by plaque assay on MDCK cells. Data is displayed as viral titer (log_10_PFU/ml) and represent the mean (± 1 SD) from triplicate samples. Data is representative of 2 independent experiments. n.s. = no significant difference, *** *p* < 0.001, two-way ANOVA followed by Bonferroni analysis.

It is well established that IAV infection of AEC results in productive viral replication, however infection of macrophages with seasonal IAV is abortive, such that viral replication is blocked and infectious viral particles are not released [27, 35]. However, the mechanisms that restrict productive IAV replication macrophages are currently unknown. Whether BST-2 can restrict release of IAV from AEC is contentious, and the role of BST-2 in modulating IAV release from macrophages has not been reported. Therefore, primary AEC and macrophages were inoculated with IAV, washed and cultured for a total of 2 hours (representative of residual inoculum) or 8, 24 or 48 hours, before titres of infectious virus were determined in cell-free supernatants by standard plaque assays. For HKx31, at 24 and 48 hours post-infection, titres of infectious virus released from BST-2-deficient AEC were significantly reduced compared to WT AEC, although this was not the case for the Brazil/78 or Sol Is/06 IAV strains, where viral release of these strains from AEC occurred independently of BST-2 (Fig 3A). For macrophages, IAV titres did not increase between 2 and 24 hours following infection with either HKx31 or Brazil/78, and this was unaffected by the presence or absence of BST-2 (Fig 3B). In these experiments, MDCK cells were included as a control to confirm that the virus inoculum could give rise to productive IAV replication and release from cells (data not shown).

### Lack of BST-2 does not alter the susceptibility of mice to IAV infection or the ability of IAV to replicate in the airways

**Fig 4:**
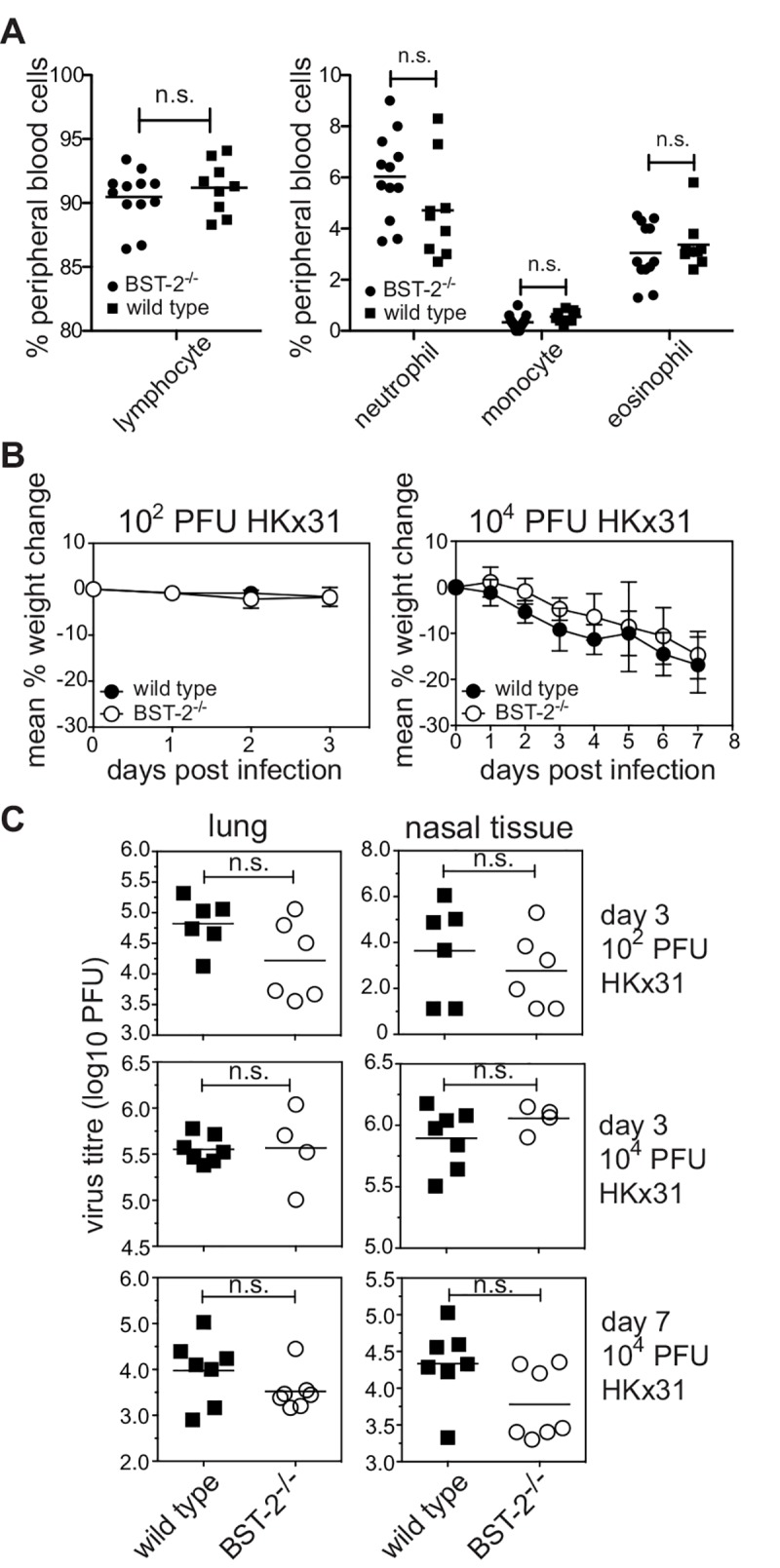
Lack of BST-2 does not alter the susceptibility of mice to IAV infection or the ability of IAV to replicate in the airways. (A) Peripheral blood from wild type and BST-2^-/-^ mice was screened by the Advia2120 automated hematology analyzer. Each symbol represents an individual mouse and the bar indicates the mean. n.s. = no significant difference, *p* = > 0.05, two way ANOVA followed by Bonferroni analysis. (B) Wild type and BST-2^-/-^ mice were infected with either 10^2^ PFU or 10^4^ PFU of HKx31 via the intranasal route. Mice were weighed daily and the results expressed as the mean percentage weight change per group (± 1 SEM) relative to original body weight. (C) Virus titres were determined in clarified homogenates prepared from lungs and nasal tissues using a standard plaque assay on MDCK cells. Symbols show titres from individual animals and horizontal bars represent the mean virus titre. n.s. = no significant difference, *p* = > 0.05, Student’s *t*-test; two-tailed.

To assess the impact of BST-2 *in vivo* we compared the susceptibility of WT BST-2 (*bst-2* ^+/+^ or *bst-2* ^+/-^) or BST-2-deficient (*bst-2* ^-/-^) mice to IAV infection *in vivo*. First, we assessed the status of BST-2-deficient mice under resting conditions. Proportions of lymphocytes, monocytes, neutrophils and eosinophils in the peripheral blood did not differ significantly between BST-2-deficient and wild type mice (Fig 4A) indicating that the absence of BST-2 did not elicit overt alterations in immune homeostasis. BST-2-deficient mice were inoculated with IAV strain HKx31 via the intranasal route. Infection of mice with this strain elicits a mild respiratory illness and weight loss is a reliable indicator of disease severity [36, 37]. Infection with HKx31 provokes a significant increase in IFNα in the respiratory tract [38] (S3 Fig) and consequently elicits conditions under which BST-2 expression is likely to be elevated. Elevated BST-2 expression has also been detected on immune cells isolated from the respiratory tract on IAV-infected mice [39]. Mice infected with 10^2^ PFU of HKx31 lost little weight by day 3 post-infection whereas infection with 10^4^ PFU resulted in progressive weight loss, however no significant differences were recorded between WT or BST-2-deficient mice (Fig 4B). IAV replication in the airways was assessed in the lung and nasal tissues at day 3 and day 7 post-infection and no significant differences were recorded between WT and BST-2-deficient mice at either time point, irrespective of inoculum dose (Fig 4C). Therefore, endogenous BST-2 does not play a major role in restricting IAV infection *in vivo*.

## Discussion

Here we have undertaken a comprehensive analysis of the role of endogenous BST-2 in restricting IAV infection by performing, to our knowledge, the first analysis of infection of BST-2-deficient primary murine cells with IAV and importantly, IAV infection of BST-2-deficient mice. Our analyses rule out a major role for BST-2 as a host molecule that restricts IAV in the mouse model of infection.

The ability of BST-2 to restrict viral infection relies on its expression by cell types permissive to infection. Analysis of surface BST-2 expression demonstrated its expression at low levels by uninfected macrophages and AEC, two cell types that are susceptible to IAV infection. In addition to inflammatory cytokines, IAV infection results in secretion of type I IFN [40], with BST-2 a type I IFN responsive gene [9]. In accordance with this, macrophages and AEC expressed increased levels of surface BST-2 in the presence of IFNα. In contrast, incubation (and infection) with IAV, increased surface BST-2 expression by macrophages, but not AEC. It is well established that IAV can induce distinct profiles of inflammatory mediators from macrophages and AEC and that macrophages induce more potent type I IFN responses than epithelial cells following IAV exposure [40]. AEC constitutively express BST-2 at high levels, but do not upregulate expression in response to IAV. We observed no major impact of BST-2 deletion on the release of IAV from AEC in single cycle replication assays. IAV may antagonize BST-2 expression in AEC [21], however the ability of IAV to antagonize BST-2 has been disputed [25] and we detect no evidence for impaired BST-2 expression following IAV infection. In summary, we observe that BST-2 is expressed by cell types of importance to IAV infection and consequently has the potential to modulate IAV infection.

Analysis of IAV infection of primary cells *in vitro* failed to support a major role for BST-2 in restricting productive virus replication and release. In AEC, the role for BST-2 varied depending on the IAV strain tested. For HKx31, BST-2 promotes, rather than inhibits, the release of newly synthesized virions from IAV-infected cells. This was not the case following infection with IAV Brazil/78 or Sol Is/06, and therefore the significance of a role for BST-2 in promoting IAV release is unclear, Regardless, we detect no evidence for a major role for BST-2 in restricting IAV release in AEC under the experimental conditions tested. It is well established that macrophages do not support productive replication of seasonal IAV [27, 35] and herein we demonstrate that the absence of BST-2 did not reverse this phenotype, despite increased expression of BST-2 following IAV infection of macrophages from WT mice. In summary, BST-2 is not acting as a dominant restriction factor for IAV in either murine AEC or macrophages.

Numerous studies have addressed the ability of BST-2 to restrict release of different viruses *in vitro*, however less is known regarding its ability to modulate viral infections *in vivo*. The availability of BST-2-deficient mice [7, 41] has allowed the impact of viral infection to be assessed in the absence of endogenous BST-2. Infection of BST-2-deficient mice with Chikungunya virus (CHIKV) results in increased viremia, in accordance with its capacity to tether CHIKV *in vitro* [42]. Similarly, infection of BST-2 deficient mice with Moloney murine leukemia virus elicits enhanced viral titers [41]. In contrast, BST-2 does not act according to its predicted role as a viral tetherin in several infection models. Infection of BST-2-deficient mice with vesicular stomatitis virus, a target of BST-2 tethering *in vitro* [6], results in reduced, rather than increased, viral titers [7]. This was also the case following infection with IBV, where BST-2-deficient mice display reduced viral titers in the lung [7]. Herein, we also report reduced levels of infectious IAV in cell supernatants from BST-2-deficient AEC, although this was only under specific experimental conditions and *in vivo* viral titers in the respiratory tract were not significantly different to those in WT animals. Increased viral titers in the presence of BST-2 is perplexing as it excludes a major role for BST-2 in restricting viral release and instead, implicates a pro-viral role for BST-2. One explanation for this is the potential role for BST-2 in enhancing, rather than inhibiting, viral entry. This is reported for cytomegalovirus, where BST-2 exerts a reverse-tethering mechanism to promote entry [43]. Note, however, that we find no evidence of BST-2 promoting infectious entry of IAV, given that BST-2-deficient AEC and/or macrophages are equally susceptible to infection compared to WT cells. How BST-2 acts to promote, rather than inhibit, IAV release remains to be determined. Regardless, our infection studies revealed similar kinetics of weight loss and viral replication in BST-2-deficient and WT mice. Therefore, in the mouse, BST-does not have a major impact on IAV infection outcomes *in vivo*.

Together, our data do not support a role for endogenous BST-2 as a major factor restricting infectious entry of primary murine IAV into target cells or in limiting the release of newly synthesized virions from infected cells. In conclusion, based on our studies in the moue model of infection, it is likely that murine IAV infection and replication can be inhibited by the (combined) action of a number of host molecules, of which BST-2 may contribute only a minor role.

## Supporting Information

S1 Checklist NC3Rs ARRIVE Guidelines Checklist. (PDF)

S1 Fig BST-2 expression. Primary macrophages isolated from *bst-2* ^+/+^ (solid black line), *bst-2* ^+/-^ (dashed line) and *bst-2* ^-/-^ (black histogram) were cultured for 24 hours with 1000 IU/ml of IFNα before analysis of cell-surface BST-2 expression by flow cytometry. Isotype control shown is for *bst-2* ^+/+^ cells and was indistinguishable from that observed for *bst-2* ^-/-^ and *bst-2* ^+/-^ cells.
(EPS)

S2 Fig Assessment of viral nucleoprotein expression in primary murine epithelial cells and macrophages following IAV infection. Primary AEC or macrophages were incubated with HKx31 at an MOI of 5 for 1 hour at 37°C, washed to remove excess virus and cultured. Monolayers were fixed with 80% vol/vol acetone at 2 or 8 hours post-infection before staining by immunofluorescence to detect newly synthesized viral NP (green) and with DAPI to stain the nucleus (blue). Similar results were also obtained with Brazil/78 for AEC and macrophages and Sol Is/06 for AEC (data not shown). Images were acquired with a Zeiss LSM700 or Olympus IX70 confocal microscope in conjunction with Zen2012 software.
(PDF)

S3 Fig Influenza A virus infection elicits IFNα in the respiratory tract. Mice were infected with 10^4^ PFU HKx31 via the intranasal route. Lungs were removed 3 days following infection. IFNα in total lung homogenates was measured by enzyme-linked immunosorbent assay. Antibodies to IFNα used for capture (22100–1) and detection (32100–1) were from PBL Assay Science (NJ, USA). Data is pooled from two independent experiments. Each symbol represents an individual mouse and the bar indicates the mean. * *p* < 0.05, Student's unpaired *t*-test.
(EPS)

## Abbreviations

AEC: alveolar epithelial cell
CHIKV: Chikungunya virus
IAV: influenza A virus
IBV: influenza B virus
MOI: multiplicity of infection
WT: wild type
