# Supplementary material for: Endogenous Murine BST-2/Tetherin Is Not a Major Restriction Factor of Influenza A Virus Infection
Source: PLoS One. 2015 Nov 13;10(11):e0142925. doi: 10.1371/journal.pone.0142925 (PMC4643895; doi:10.1371/journal.pone.0142925)
Supplement: S2 Fig — Primary AEC or macrophages were incubated with HKx31 at an MOI of 5 for 1 hour at 37°C, washed to remove excess virus and cultured. Monolayers were fixed with 80% vol/vol acetone at 2 or 8 hours post-infection before staining by immunofluorescence to detect newly synthesized viral NP (green) and with DAPI to stain the nucleus (blue). Similar results were also obtained with Brazil/78 for AEC and macrophages and Sol Is/06 for AEC (data not shown). Images were acquired with a Zeiss LSM700 or Olympus IX70 confocal microscope in conjunction with Zen2012 software. (PDF) [file pone.0142925.s003.pdf]

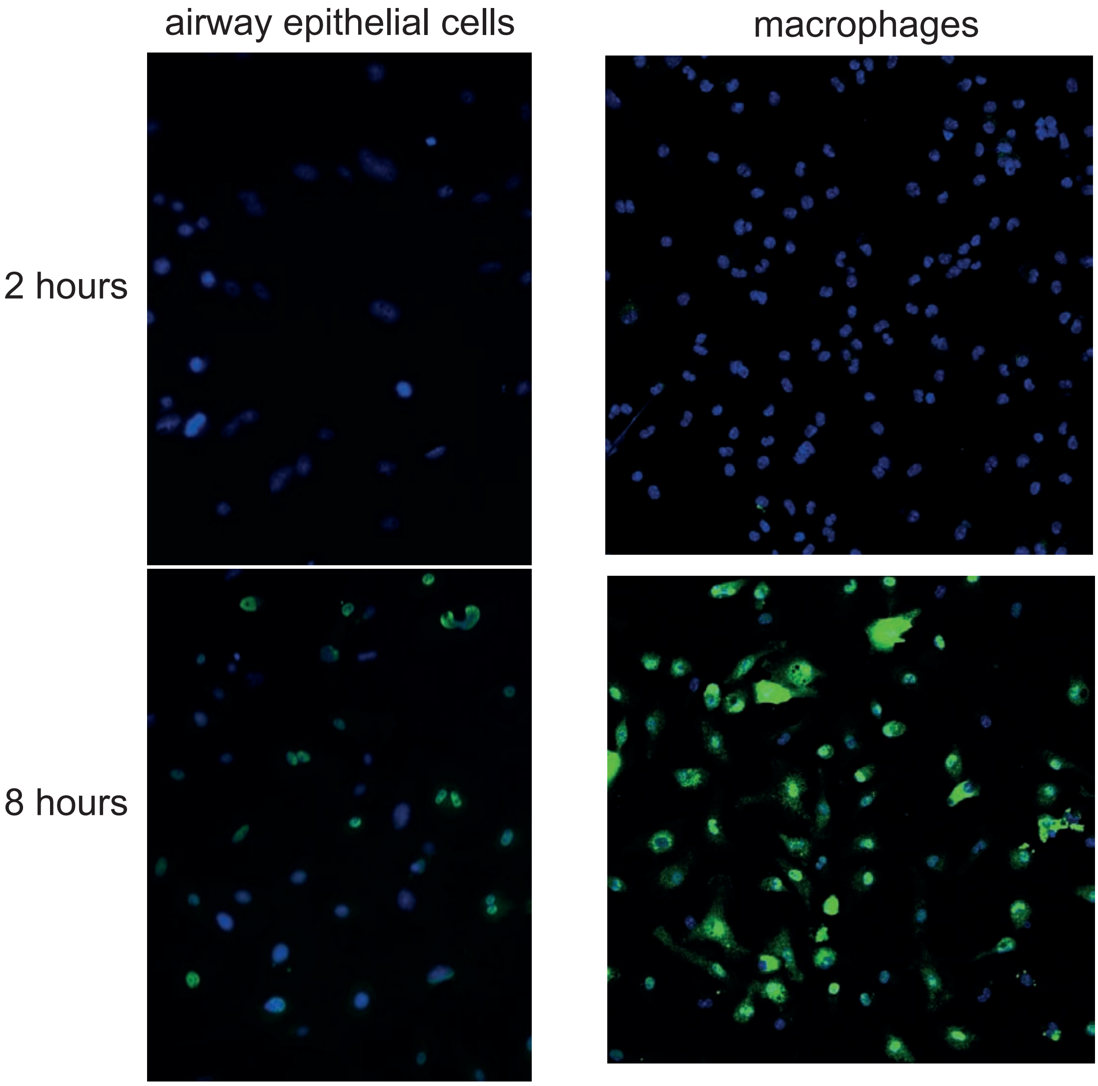

S2 Fig. Assessment of viral nucleoprotein expression in primary murine epithelial cells and macrophages following IAV infection.
